# Superior outcomes of kidney transplantation compared with dialysis: An optimal matched analysis of a national population-based cohort study between 2005 and 2008 in Korea

**DOI:** 10.1097/MD.0000000000004352

**Published:** 2016-08-19

**Authors:** Kyung Don Yoo, Clara Tammy Kim, Myoung-Hee Kim, Junhyug Noh, Gunhee Kim, Ho Kim, Jung Nam An, Jae Yoon Park, Hyunjeong Cho, Kyoung Hoon Kim, Hyunwook Kim, Dong-Ryeol Ryu, Dong Ki Kim, Chun Soo Lim, Yon Su Kim, Jung Pyo Lee

**Affiliations:** a Department of Internal Medicine, Division of Nephrology, Dongguk University Medical Center; b School of Public Health, Seoul National University, Seoul; c Department of Dental Hygiene, College of Health Science, Eulji University, Daejeon; d College of Engineering, Seoul National University; e Department of Internal Medicine, Seoul National University Boramae Medical Center; f Department of Internal Medicine, Seoul National University College of Medicine; g Department of Public Health, Graduate School, Korea University, Seoul; h Department of Internal Medicine, Wonkwang University College of Medicine, Sanbon Hospital, Gyeonggi-do; i Department of Internal Medicine, School of Medicine, Ewha Womans University, Seoul, Republic of Korea.

**Keywords:** dialysis, kidney transplantation, mortality

## Abstract

Data regarding kidney transplantation (KT) and dialysis outcomes are rare in Asian populations. In the present study, we evaluated the clinical outcomes associated with KT using claims data from the Korean national public health insurance program. Among the 35,418 adult patients with incident dialysis treated between 2005 and 2008 in Korea, 1539 underwent KT. An optimal balanced risk set matching was attempted to compare the transplant group with the control group in terms of the overall survival and major adverse cardiac event–free survival. Before matching, the dialysis group was older and had more comorbidities. After matching, there were no differences in age, sex, dialysis modalities, or comorbidities. Patient survival was significantly better in the transplant group than in the matched control group (*P* < 0.001). In addition, the transplant group showed better major adverse cardiac event–free survival than the dialysis group (*P* < 0.001; hazard ratio, 0.49; 95% confidence interval, 0.32–0.75). Korean patients with incident dialysis who underwent long-term dialysis had significantly more cardiovascular events and higher all-cause mortality rates than those who underwent KT. Thus, KT should be more actively recommended in Korean populations.

## Abstract 2

Supplemental Digital Content is available in the text

## 1 Introduction

End-stage renal disease (ESRD) increases the incidence of comorbidities and the mortality rate, which are difficult to prevent.^[1]^ However, despite interest in the burden of this disease, the incidence of ESRD is steadily increasing annually.^[2,3]^ Kidney transplantation (KT) is the best treatment for patients with ESRD in terms of increasing the survival rate, reducing complications, and improving quality of life.^[4–6]^ The degree of clinical benefit associated with KT has recently become important.^[7]^ In Asia, however, there are insufficient data regarding KT and dialysis outcomes. The benefit of KT differs according to sociocultural, racial, and health insurance coverage factors.^[8]^ Therefore, ethnic differences should be considered because they can affect clinical KT outcomes.^[8]^ Thus, investigations into KT versus dialysis outcomes in Asian populations remain important.

Cardiovascular disease is a leading cause of mortality in patients with ESRD, even among those undergoing transplantation.^[9,10]^ The long-term use of immunosuppressive agents may induce hypertension, diabetes, and dyslipidemia.^[11]^ Thus, there are several concerns regarding whether KT can achieve a reduction in cardiovascular events.^[12]^ Moreover, emerging evidence regarding racial differences in the cardiovascular outcomes of KT recipients cannot be explained by traditional risk factors alone, even among the Asian population.^[13,14]^ Therefore, the effect of KT on cardiovascular outcomes remains to be clarified, especially in Asian recipients.

A national population-based cohort study used data from the Korea Health Insurance Review and Assessment Service (HIRA). This cohort is a complete enumeration survey of the entire population of Korea because all citizens are mandatorily covered by the government-initiated public health insurance program^[15]^; therefore, using this cohort should allow for more representative transplant outcomes of Asians, eliminating any potential environmental, socioeconomic, and racial differences. Using this cohort, we recently reported that the overall mortality rate of patients with incident peritoneal dialysis (PD) was consistently higher than that of patients on hemodialysis (HD).^[16]^

There have been several methodological issues about comparing maintenance dialysis and KT in terms of patients requiring renal replacement therapy.^[7]^ In the present study, we aimed to investigate data for all-cause mortality and cardiovascular outcomes between KT patients and patients on dialysis using an optimal balanced risk set matching (OBM) analysis of a national population-based cohort in South Korea. We attempted to resolve the methodological concerns about maintenance dialysis versus undergoing transplant in terms of clinical outcomes using a newly developed matching method.

## 2 Materials and methods

### 2.1 Data source and study participants

In Korea, the National Health Security System (HSS) is a mandatory social insurance program; it is composed of the National Health Insurance (NHI) and Medical Aid and is overseen by the Ministry of Health and Welfare. Under this system, all data are stored and managed in the National Health Insurance Claims Database, which contains all the information about reimbursements under the fee-for-service system (NHI) or per diem system (Medical Aid) for patients on dialysis.^[17,18]^ Using the national population-based cohort from the Korea HIRA, we obtained data for all patients who underwent KT and those who underwent incident dialysis between January 1, 2005, and December 31, 2008, in Korea. These data recently became an open-access research resource to help Korean researchers perform comprehensive public health studies on patients on dialysis.^[16,19,20]^

**Figure 1:**
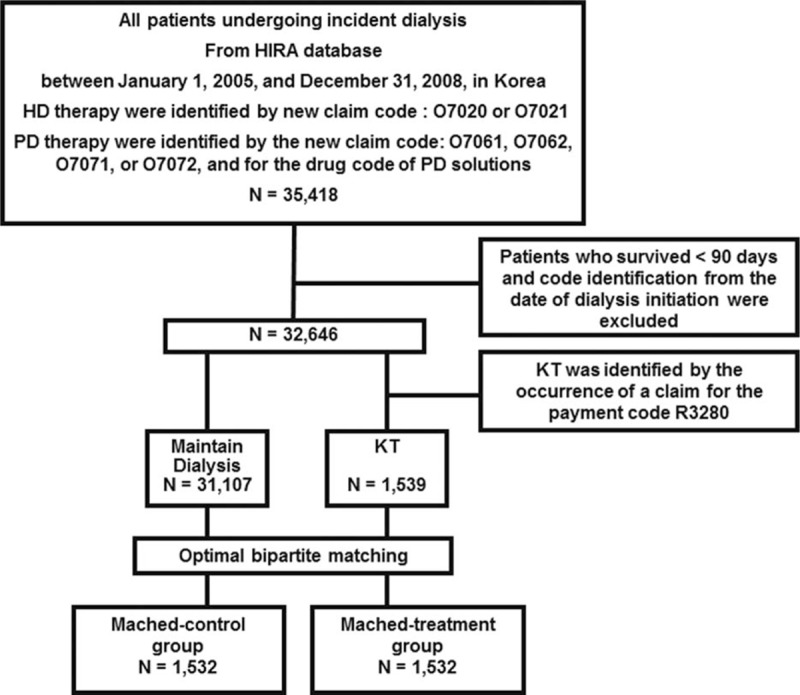
Study flowchart. HD = hemodialysis, HIRA = Korea Health Insurance Review and Assessment Service, KT = kidney transplantation, PD = peritoneal dialysis.

Briefly, KT was identified by the occurrence of a claim for the payment code R3280.^[15,16]^ The methods used to identify patients on dialysis and their comorbidities using the HIRA database have been described previously.^[16,19]^ Comorbidities were identified by reviewing patients’ medical history during the year before the initiation of dialysis therapy. We excluded 2772 patients who survived for <90 days from the date of dialysis initiation (Fig. 1). The present study was performed in accordance with the Helsinki Declaration and was approved by the institutional review board of Seoul National University Hospital (no.: 1407-146-597).

### 2.2 Outcomes

The primary outcome was the cumulative overall survival rate during the observation period. The secondary outcome of this study was the occurrence of major adverse cardiac events (MACE) during the follow-up, which was defined as a composite of the incidence of nonfatal myocardial infarction (MI), hemorrhagic and ischemic stroke, and coronary artery bypass graft surgery or percutaneous coronary intervention.

### 2.3 Statistical analysis

In this study, we considered 2 methods for analysis to compare 2 groups to account for the fact that patients with ESRD switched from the dialysis group to the transplantation group during the follow-up, and patients with ESRD had different baseline characteristics between the transplant and dialysis groups. If patients with ESRD at the time of kidney transplant were directly compared to patients with ESRD on dialysis, the time to the dialysis exposure status among patients who received a kidney transplant would not be addressed. The term “immortal time bias” has been used to describe this circumstance.^[21]^ One of the proper ways to account for this matter is to use a time-dependent hazard model. It considers the time-varying nature of the exposure status and minimizes the possibility of immortal time bias from time-varying exposures.^[5,21]^

The other issue is adjusting the baseline characteristics between the treatment and control groups. Observational studies are a yielding way of assessing treatment effects in a general population with low cost. However, observational studies could not select the patient and control group under restricted clinical supervision, so they may cause lack of a control group. These limitations may lead to potential bias. Propensity score matching (PSM) is one of the popular methods to solve this issue. However, with conventional PSM, the control group should be matched only with patients who do not receive a transplant; therefore, it is possible that the groups will not be similar with respect to the relevant prognostic factors for comparison with kidney transplant recipients (KTRs). For example, transplant candidates are generally considered to have fewer risk characteristics than patients undergoing maintenance dialysis. Therefore, conventional matching would overestimate the survival advantage associated with receiving a kidney transplant.

Thus, we accounted for these 2 issues by using the time-dependent PSM method, that is, OBM. This method considers the time-dependent exposure and balances covariates of the 2 groups.^[22–24]^ The time-dependent propensity score is computed for each patient using the treatment hazard, and each treated patient is then matched with an alive, uncensored, and not-yet-treated patient with a similar propensity score. More details about the estimation of the propensity score using a Cox proportional model are described in previous reports.^[22–24]^

The major differences between risk set matching and conventional matching are the following: risk set matching uses the risk of receiving treatment, which is related to the Cox hazard model. The time-varying hazard of treatment works like the propensity score. Second, in risk set matching, a patient treated at time Ti is matched to a patient not yet treated at time Ti rather than to a patient who never received a transplant. This means that a patient who undergoes transplantation at time Ti can enter the study as a treated patient or as a not-yet-treated control for the other transplant patient prior to time Ti.

We included patients’ characteristics such as age, sex, and the dialysis modality; the HSS, which is composed of the NHI and Medical Aid data; comorbidities such as diabetes mellitus (DM), MI, congestive heart failure (CHF), peripheral vascular disease (PVD), cerebrovascular disease (CVD), chronic obstructive pulmonary disease (COPD), peptic ulcer disease, liver disease, and any cancer; and the Charlson Comorbidity Index (CCI) to estimate the time-dependent propensity score.^[25]^ Matching was performed for each of the risk sets. When there was 1 treated patient or multiple treated patients in the risk set, controls who were closest in terms of the time-dependent propensity score were chosen. The matched subjects were removed from the next risk sets. The same process continued and was repeated with the next risk set. The matching process stopped when there were no more treated patients in the risk set. Our methods were easy to implement in R, and the code is presented in Supplementary Table 1.

In the present study, continuous and categorical variables were compared between the transplant and control groups using the *t* test and χ^2^ test, respectively. Standardized differences were also used to compare baseline characteristics between the 2 groups before and after OBM.^[26]^

Kaplan–Meier survival curves were estimated for the transplant and control groups after OBM. The Peto and Peto modification of the Gehan–Wilcoxon test was used to compare the Kaplan–Meier survival curves from the matched dataset.

For the multivariate hazard model, we did not include CCI as an adjusting covariate because multicollinearity issues arise when too many variables are added to the model. We performed a stratified subgroup analysis by age (18–39, 40–49, 50–59, >60 years), sex, HSS, the dialysis type, and 9 comorbidities (DM, MI, CHF, PVD, CVD, COPD, peptic ulcer disease, liver disease, and any cancer). Subgroup analyses were used to evaluate the consistency of treatment across multiple groups. We performed an interaction test to confirm the modifying effects of each variable. However, the results of the subgroup analyses may need to be interpreted with caution because of the potential type 1 error that can occur with multiple comparisons.^[27–30]^ All of the statistical analyses were conducted using SAS (version 9.3; SAS Institute, Inc, Cary, NC) and R version 2.14 for Windows (http://cran.r-project.org/). PSM was performed using the optmatch package in R.^[31]^

### 2.4 Sensitivity analysis

Our data do not include KT waiting-list information and could not separate the deceased donor KTRs from the living donor KTRs because the HIRA data do not include information about the donor type. The comparison of the clinical outcomes of transplant recipients with those of patients in the transplant waiting list has been considered appropriate.^[5,9]^ There are several reasons why previous studies did not compare the transplant group with an all ESRD patient group. One of the main reasons is the biased predictions. If all patients with ESRD are set as the control group to the transplant treatment group, positive effects of transplantation may be overestimated.^[5,9]^

To overcome this limitation, we conducted several additional analyses. We used the Korean Network for Organ Sharing (KONOS) data for these analyses.^[17]^ First, we compared the survival between living donor kidney transplant, deceased donor kidney transplant, and KT wait-listed patients in the KONOS data. Second, we compared the survival results between the wait-listed patients in the KONOS data and the matched control patients in the HIRA data. The purpose of the sensitivity analysis was to match the baseline characteristics of the matched control group (who did not undergo transplantation) from the HIRA data to those of the KT wait-listed patients from the KONOS data in Korean patients with ESRD.^[17]^ Furthermore, we conducted analyses using the Clinical Research Center (CRC) for ESRD (NCT00931970) database to resolve the validity of MACE of the matched control group in our study cohort.

## 3 Results

### 3.1 Baseline characteristics of the study population before and after optimal balanced risk set matching

Patients’ baseline characteristics were compared between the transplant and dialysis groups. In total, 1539 subjects undergoing KT between 2005 and 2008 were included in the transplant group (Table 1).

**Table 1:**
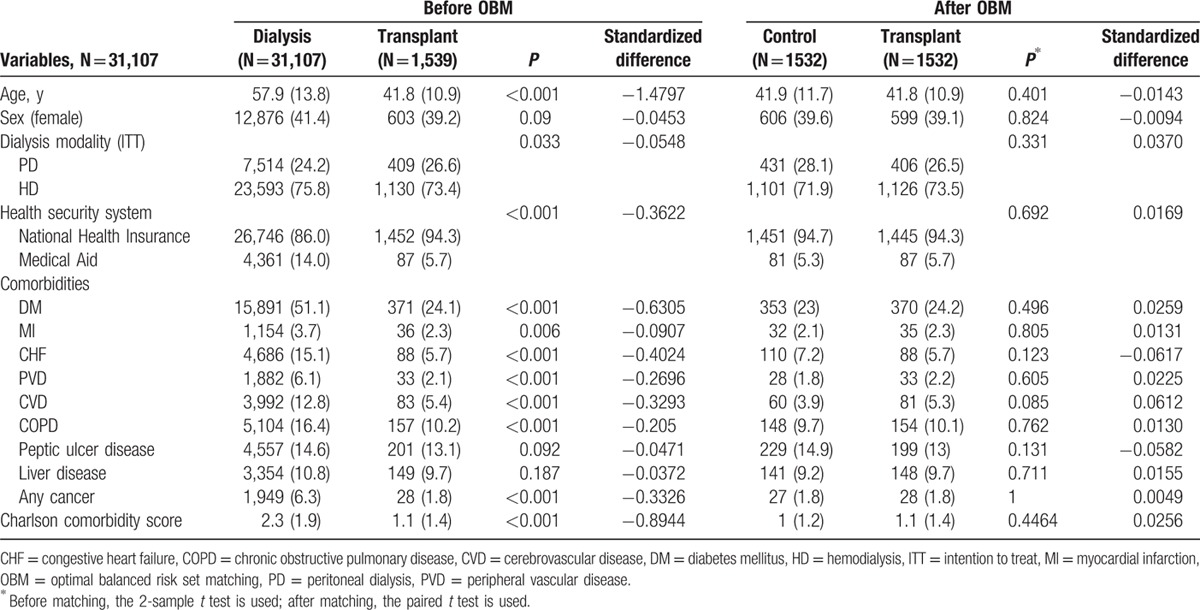
Patients’ characteristics before and after optimal balanced risk set matching between the dialysis group and the transplant group.

Before OBM, there were significant differences in age, dialysis modality, insurance type (NHI vs Medical Aid), and comorbidities between the groups. The mean patient age was 57.9 years in the dialysis group and 41.8 years in the transplant group before OBM (*P* < 0.001). Patients undergoing PD had a greater likelihood of undergoing KT than those who underwent HD (26.6% vs 24.2%; *P* = 0.033). The dialysis group had a larger proportion of patients with Medical Aid coverage (14.0% vs 5.7%; *P* < 0.001). In addition, the dialysis group had a greater number of comorbidities such as diabetes, MI, CHF, PVD, CVD, COPD, and any cancer.

After OBM, there were no differences in any of the variables, including age, sex, the dialysis modalities, and comorbidities. The mean patient age was 41.8 years in both groups (*P* = 0.401). Table 1 shows that the standardized difference value decreased after OBM.

### 3.2 Comparisons of all-cause mortality and cardiovascular morbidities between the transplant and matched control groups

**Figure 2:**
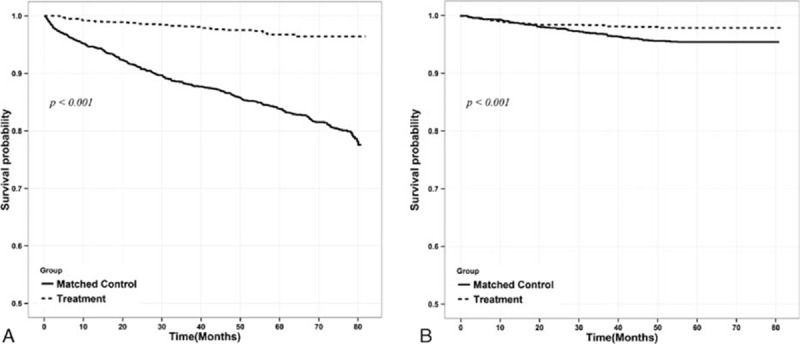
Comparison of cumulative survival rate in the control group and transplant group for overall survival rate (A) and major adverse cardiac events (MACE) (B) after optimal balanced risk set matching. (A) Kaplan–Meier curves for the matched population including those in the control group (solid line) versus transplantation group (dashed line); (B) Kaplan–Meier curves for MACE in the matched population (solid line) versus transplantation group (dashed line). MACE: incidence of nonfatal myocardial infarction, hemorrhagic and ischemic stroke, coronary artery bypass graft surgery, or percutaneous coronary intervention.

All-cause mortality and MACE were compared between the groups after performing OBM using the Gehan–Wilcoxon test. The transplant group had a 2.5% crude all-cause mortality rate (38 of 1532 patients) during the follow-up period (median follow-up, 26.5 months), whereas the control group had a 19.2% crude all-cause mortality rate (294 of 1532 patients). The transplant group had a significantly better overall survival rate on the Kaplan–Meier curve (*P* < 0.001; Fig. 2A). In multivariate analysis, the transplant group had better overall survival than the control group after adjusting for confounding factors (hazard ratio [HR], 0.15; 95% confidence interval [CI], 0.11–0.22; *P* < 0.001) (Table 2).

**Table 2:**
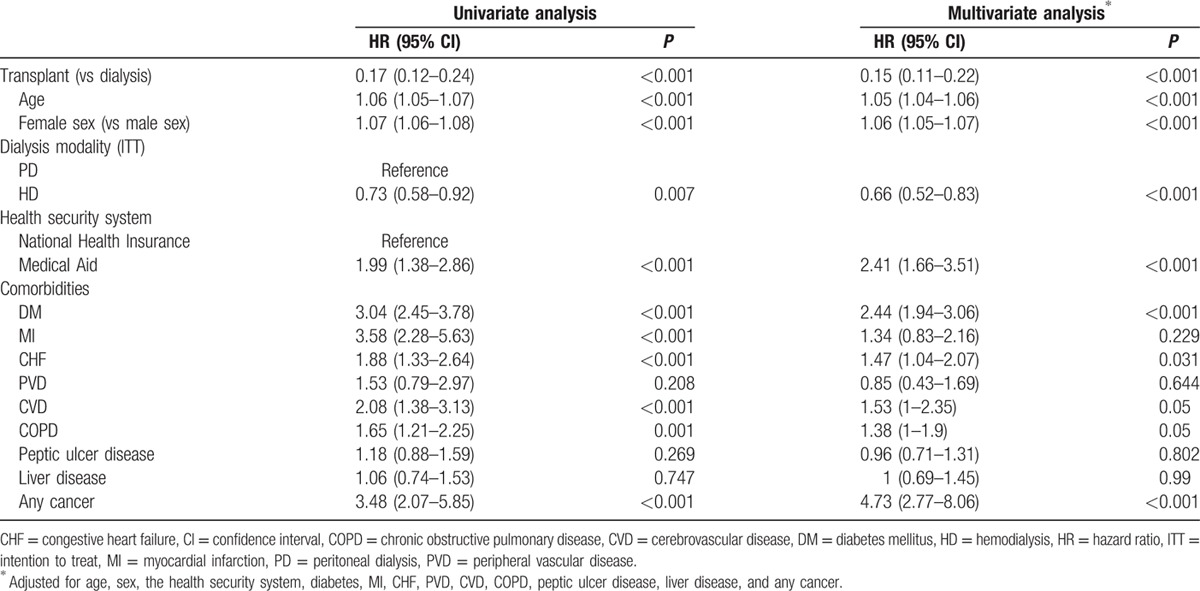
Results from the Cox hazard model for all-cause mortality using univariate and multivariate analyses after optimal balanced risk set matching.

Transplantation had the benefit of greater cardiovascular event-free survival than maintenance dialysis, after adjusting for other confounding variables. MACE occurred in 30 patients in the transplant group and in 70 in the control group during the follow-up period. Survival analysis showed that the transplantation group had a better MACE-free survival rate (*P* < 0.001; Fig. 2B). In Cox regression analysis, the transplant group had better MACE-free survival than the control group after adjusting for age, sex, the dialysis modality, the health insurance type, and comorbidities (HR, 0.49; 95% CI, 0.32–0.75; *P* < 0.001) (Table 3).

**Table 3:**
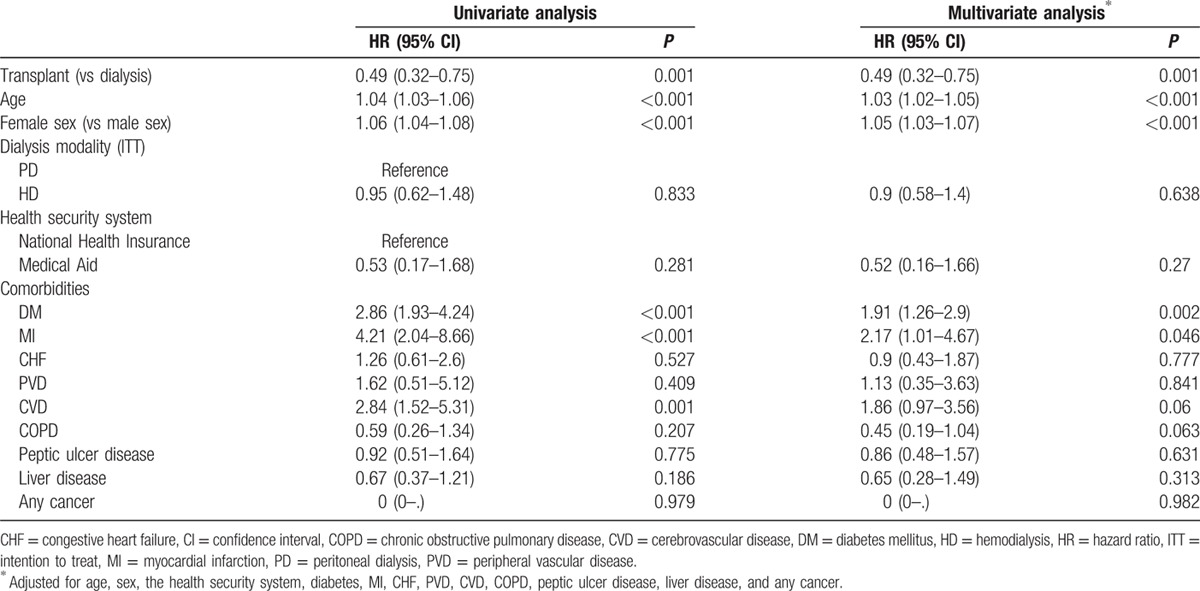
Results from the Cox hazard model for major cardiovascular adverse events using univariate and multivariate analyses after optimal balanced risk set matching.

### 3.3 Subgroup analyses according to baseline characteristics after optimal balanced risk set matching

**Figure 3:**
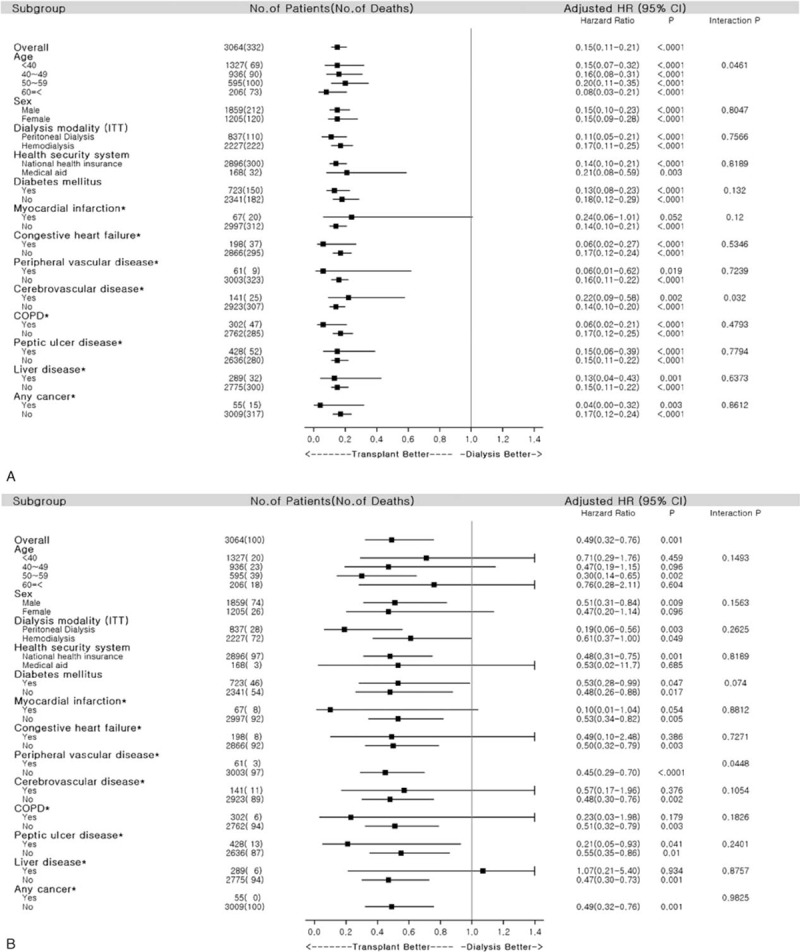
Comparison of adjusted hazard ratios (HRs) for subgroups. (A) Adjusted HR of each subgroup for all-cause mortality; (B) adjusted HR of each subgroup for major adverse cardiac events (MACE). The adjusted covariates included age, sex, dialysis modality, health security system (HSS), and comorbidities (diabetes mellitus, myocardial infarction, congestive heart failure, peripheral vascular disease, cerebrovascular disease, chronic obstructive pulmonary disease, peptic ulcer disease, liver disease, and any type of cancer) for each subgroup (excluding own). (∗) The adjusted covariates included age, sex, dialysis modality, HSS, and Charlson comorbidity score for each subgroup. MACE: incidence of nonfatal myocardial infarction, hemorrhagic and ischemic stroke, coronary artery bypass graft surgery, or percutaneous coronary intervention. CI = confidence interval, COPD = chronic obstructive pulmonary disease, HR = hazard ratio, ITT = intention to treat.

Thereafter, we performed a subgroup analysis to confirm the clinical benefit of KT in the different subgroups. We divided the study population into subgroups by age, the dialysis modality, sex, the health insurance type, and comorbidities (Fig. 3A). When multivariate analysis was performed for all-cause mortality by age group, the transplant group had better overall survival than the control group in all age groups. In elderly patients (≥60 years), KT improved overall survival (HR, 0.08; 95% CI, 0.03–0.21; *P* < 0.001). In the other subgroups, transplantation showed a greater survival benefit compared to the control after adjusting for confounding factors. KT reduced all-cause mortality in patients with DM (HR, 0.13; 95% CI, 0.08–0.23; *P* < 0.001). Interaction tests were marginal by age group.

Subgroup analysis for MACE-free survival (Fig. 3B) showed that the transplant group had better MACE-free survival than the control group in most of the subgroups. Interaction tests were not significant in any subgroup analysis.

### 3.4 Results of sensitivity analysis

The additional analysis using data from the KONOS database resulted in a much lower cumulative survival for the wait-listed patients than both the living donor and deceased donor patients with KT (Supplemental Fig. 1). There was no difference in the cumulative survival rates between wait-listed patients and the matched control group (who did not undergo transplantation) after PSM in the additional survival analysis using the KONOS database and HIRA data (Supplemental Fig. 2).

When comparing the incidence of MACE (data not shown) and the MACE-free survival rate between patients from the CRC for ESRD database and matched control group in our study cohort (HIRA data) (Supplemental Fig. 3), there was no difference in the cardiovascular outcomes between the groups. The CRC for ESRD is a prospective cohort; therefore, the patients have not been followed up to date. This should be considered in the interpretation of our additional analysis.

## 4 Discussion

This is the first Asian national population-based study to evaluate the direct clinical benefits of KT in patients with incident dialysis using data from the national database of a public health insurance program. The present study reports the characteristics of all KTRs among patients with incident dialysis between 2005 and 2008 in Korea, and the results advocate a survival benefit of KT. In contrast, Korean patients with incident dialysis who underwent long-term dialysis had significantly more cardiovascular events and higher all-cause mortality rates.

Although KT has better clinical and socioeconomic benefits than maintenance dialysis, these findings should be interpreted with caution.^[7]^ The benefits of KT may have resulted from selection bias because patients undergoing maintenance dialysis without KT tend to have a greater number of and more severe comorbidities than those undergoing KT. It is difficult to conduct a randomized control study comparing dialysis with transplantation due to ethical issues. Moreover, several methodological concerns about the comparison of maintenance dialysis and KT have been raised.^[7]^ To overcome differences in the baseline characteristics, many other studies have used KT wait-listed patients as study controls.^[5,32–37]^

As mentioned in Section 2.4, we could not directly compare KTRs to patients on dialysis on the waiting list because HIRA data do not indicate if patients are registered on a waiting list, which could be considered a limitation of the study. Nonetheless, we suggest that the optimal matched control group have similar baseline characteristics to patients who are candidates for KT. Considering the time-varying exposure that occurs in an observational study setting, OBM was conducted in the present study instead of conventional 2-group greedy matching.^[22–24]^ Lu et al presented a risk set matching method that uses a hazard of receiving treatment. The time-varying cox hazard is as follows: 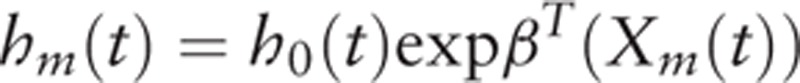


It is used to estimate the hazard of being treated at a certain time point for each patient.^[22,23]^ If *M* is the number of patients entered into the study, *m* = 1, 2, 3, …, *M*, where patient *m* is enrolled in the study at certain time point and may be treated at any time or not at all. *X*_*m*_(*t*) is the vector of covariates for patient *m* at time *t*. The hazard for patient *m* at time *t* is *h*_*m*_(*t*).

Because of the characteristics of the HIRA data, there are time-fixed variables that consider baseline characteristics for all covariates, except for the time point of the transplant, and newly treated patients were matched with patients not yet treated by using baseline covariates via the hazard of receiving the treatment. More details about the estimation of the propensity score using a Cox proportional model are described by Lu et al.^[22,23]^

In the present study, we also found that patients who underwent PD required KT more often than patients on HD (*P* = 0.033) (Table 1). PD was recently suggested as a bridge therapy before transplantation.^[38–40]^ Schwenger et al reported that pretransplant PD can reduce all-cause mortality by 10% compared with pretransplant HD.^[39]^ This may be one of the reasons why we found poorer outcomes with maintenance PD than with maintenance HD in our recent study.^[16]^ Another interesting finding of the present study was that patients with a low socioeconomic status (SES) who were covered by Medical Aid had a lower chance of undergoing KT. In addition, a low SES was significantly associated with a 2.41-fold poorer overall survival (Table 2). In studies regarding the association between ethnic background and transplant outcomes conducted in Western countries, household income disparity was a primary confounder and/or mediator.^[7,8,41]^ Although the association between transplant outcomes and SES remains controversial,^[7,8]^ maintaining the HSS can affect the outcomes of transplant and dialysis patients.^[42]^

A systematic review reported that KT reduces the risk of death compared with patients on dialysis on the transplant waiting list, with an HR of 0.16 to 0.73 in 94% of patients according to the reviewed studies.^[7]^ Our study showed that transplantation improved overall survival and MACE-free survival compared to dialysis without KT (HR, 0.15 and 0.49, respectively). The benefits appear to be better than those observed in previous studies. This can be explained by the less severe comorbidities in the KTRs in Korea than those in previous studies.^[43]^ A high number of comorbidities, according to the CCI, are closely correlated to posttransplant patient survival. Wu et al reported that a CCI ≥5 increased the risk of death by 2.88-fold.^[43]^ In the present study, the mean CCI in the transplant group was 1.1 and that in the dialysis group was 2.3, whereas that in transplant recipients in the study by Wu et al was 3.2. Moreover, the proportion of living-donor KTs in Korea was higher than those in Western countries, although the data were not available in this study.^[17]^ In the present study, diabetes was the most common comorbid condition (24.1%), which was similar to the findings of Wu et al. Additionally, diabetes, CVD, and any cancer were independent prognostic factors for mortality. These findings are consistent with previous studies that suggest that diabetes^[44]^ or CVD^[45,46]^ worsened survival rates compared to those of patients without comorbidities.

We assessed MACE-free survival, which included cardiovascular events and cerebrovascular events (i.e., hemorrhagic and ischemic stroke). In KTRs, the most common cause of death is cardiovascular disease, accounting for 50% to 60% of such cases.^[47]^ Cardiovascular-related deaths commonly occur in diabetic KTRs, whereas other causes such as tumors and infections are associated with nondiabetic recipients.^[48]^ The deaths of approximately half of the cases with cardiovascular disease were associated with CVD.^[49]^ The main concern with using claims data is how to define the incidence of MACE, and the accuracy of diagnoses, especially for cardiovascular events using claims data, has been addressed previously. The prospective cohort in the Korean Heart Study validated an acute MI diagnosis of 71.4% of patients using claims data.^[50]^ Previously, Park et al reported an 83% accuracy of diagnosing CVDs using the claims data of 115,600 people.^[51]^ Our study showed better MACE-free survival compared with other studies (HR, 0.49; CI, 0.32–0.75). Although several earlier studies have confirmed the cardiovascular benefits of KT in various clinical phenotypes, including diabetic nephropathy and lupus nephritis,^[7,52–57]^ they included few Asian patients. To our knowledge, the present study is the largest study to show the cardiovascular benefit of KT in an East Asian population.

A subgroup analysis was additionally performed to clarify the benefit of KT among high-risk KTRs. Patients in the transplant group had better survival than those in the dialysis group among all age groups. The posttransplant outcomes of elderly Asians are not well known. We expect that results from stratification analysis will aid in our understating of elderly KTRs. Moreover, certain comorbidities such as diabetes affected the patients in different ways due to ethnic heterogeneity. Although diabetes remains a risk factor for poor outcomes in KTRs, there are no reports in Asian KTRs with diabetic nephropathy.^[48,52,58]^ The present study showed that patients in the transplant group had better overall survival and cardiovascular outcomes than those in the dialysis group in most Asian subpopulations with high-risk comorbidities, including old age and diabetes.

Our data had several limitations, including those mentioned previously.^[16]^ First, although immunological and nonimmunological factors can affect the clinical outcomes of kidney transplant, we could not obtain data on immunological factors such as the donor age, donor sex, donor type, human leukocyte antigen mismatching, and immunosuppressive medication. Second, graft outcomes were also unavailable. Third, the observation period was relatively short. Finally, our data do not include KT waiting-list information. To overcome this limitation, we conducted several validation analyses.

In conclusion, among Korean patients with incident dialysis, those who underwent KT showed significantly improved overall survival and cardiovascular outcomes compared with those who remained on dialysis. Hence, KT should be more actively recommended in the Korean population.

## Supplementary Material

Supplemental Digital Content
