# Supplementary material for: Superior outcomes of kidney transplantation compared with dialysis: An optimal matched analysis of a national population-based cohort study between 2005 and 2008 in Korea
Source: Medicine (Baltimore). 2016 Aug 19;95(33):e4352. doi: 10.1097/MD.0000000000004352 (PMC5370789; doi:10.1097/MD.0000000000004352)

**Supplemet Table 1. Optimal risk set matching process in MatchIt Package in R**

| **Code sheet** |
| --- |
| install.packages("optmatch",repos=c("http://rstudio.org/_packages", "http://cran.rstudio.com"))  library(optmatch)  install.packages("survival",repos=c("http://rstudio.org/_packages", "http://cran.rstudio.com"))  library(survival)  args <- commandArgs(TRUE)  #Mode <- 0 # 0 if permit match with late transplant, otherwise 1  Mode <- as.numeric(args[1])  wait.unit <- as.numeric(args[2])  Mode <- 0  wait.unit <- 1  setwd("~/150711_optimalMatching")  data <- read.csv('final07_90days4.csv')  head(data)  str(data)  table(data$transplant)  data$dialysis_startdate <- as.Date(data$dialysis_startdate)  data$newonset <- as.Date(data$newonset)  data$end_followup <- as.Date(data$end_followup)  data[data$dial_to_trans_dur == ".", "dial_to_trans_dur"] <- NA  data$dial_to_trans_dur <- as.numeric(as.character(data$dial_to_trans_dur))  data.0 <- data[data$transplant==0,]  data[data$transplant==0, "dial_to_trans_dur"] <- as.numeric(data.0$end_followup - data.0$dialysis_startdate)  fit <- coxph(Surv(dial_to_trans_dur, transplant) ~ hss + age_new + sex + como1 + ITT_hemo  + como2_mi + como3_hf + como4_pvd + como5_cvd + como6_copd + como7_pud  + Charlson_score + como8_liver + como_cancer, data=data)  n <- dim(data)[1]  n.1 <- table(data$transplant)["1"][1]  hzrd <- predict(fit, newdata=data, type="lp")  if(wait.unit==1){  data$dial_to_trans_dur <- floor(data$dial_to_trans_dur/30.5)  }  hzrd.data <- as.data.frame(cbind(data$obs2, hzrd, data$transplant, data$dial_to_trans_dur))  colnames(hzrd.data) <- c("obs", "hazard", "transplant", "wait")  rownames(hzrd.data) <- hzrd.data$obs  head(hzrd.data)  hzrd.1 <- hzrd.data[hzrd.data$transplant==1,]  hzrd.0 <- hzrd.data[hzrd.data$transplant==0,]  rm(list=setdiff(ls(), c("hzrd.data", "hzrd.0", "hzrd.1", "Mode", "wait.unit")))  gc()  match.results <- c()  remain.1 <- hzrd.1  remain.0 <- hzrd.0  cat("Mode = ", Mode, "/ Time Unit = ", wait.unit, "\n")  while(dim(remain.1)[1] > 0){  t <- min(unique(remain.1$wait))  cat("Time = ", t, "\n")  set.treat <- remain.1[remain.1$wait==t,]  if(Mode==0){  set.risk <- rbind(remain.1[remain.1$wait>t,], remain.0)  }else{  set.risk <- remain.0  }  set.treat$risk <- 1  set.risk$risk <- 0  data.t <- rbind(set.treat, set.risk)  match_on.result <- match_on(risk~hazard, data=data.t, method="euclidean")  dim(match_on.result)  pm <- pairmatch(match_on.result, data=data.t)  groups <- names(table(pm))  get.pair <- function(g){  names(pm)[which(pm==g)]  }    match.pair <- t(sapply(groups, get.pair))  colnames(match.pair) <- c("Group1.ID", "Group2.ID")  # match.results <- cbind(match.results, match.pair)    Group1.ID <- as.numeric(as.character(match.pair[,"Group1.ID"]))  Group2.ID <- as.numeric(as.character(match.pair[,"Group2.ID"]))    get.transplant <- function(id){  data.t[data.t$obs == id, c("transplant", "wait")]  }    Group1.transplant <- t(sapply(Group1.ID, get.transplant))  Group2.transplant <- t(sapply(Group2.ID, get.transplant))    match.pair <- cbind(Group1.ID, Group1.transplant, Group2.ID, Group2.transplant)  match.results <- rbind(match.results, match.pair)    id.remove <- c(Group1.ID, Group2.ID)  remain.1 <- remain.1[!(remain.1$obs %in% id.remove),]  remain.0 <- remain.0[!(remain.0$obs %in% id.remove),]  }  colnames(match.results) <- c("Group1.ID", "Group1.transplant", "Group1.wait", "Group2.ID", "Group2.transplant", "Group2.wait")  fileName = paste("SequentialMatch_Mode", as.character(Mode), "_Unit", as.character(wait.unit), ".csv", sep="")  write.table(match.results, fileName, sep=",", row.names=FALSE, col.names=TRUE) |

References

1. Lu B (2005) Propensity score matching with time-dependent covariates. Biometrics 61: 721-728.

2. Lu B, Greevy R, Xu X, Beck C (2011) Optimal Nonbipartite Matching and Its Statistical Applications. The American Statistician 65: 21-30.

3. Rosenbaum PR (2002) Observational studies: Springer.

4. Li YP, Propert KJ, Rosenbaum PR (2001) Balanced Risk Set Matching. Journal of the American Statistical Association 96: 870-882.

5. Ho DE. MatchIt: nonparametric preprocessing for parametric causal inference,

Departments of Mental Health and Biostatistics, Johns Hopkins Bloomberg School of Public

**Supplement Figure 1.** Comparison of the cumulative survival rate between the wait-listed patients and kidney transplant patients in Korea using the Korean Network for Organ Sharing database (KONOS)


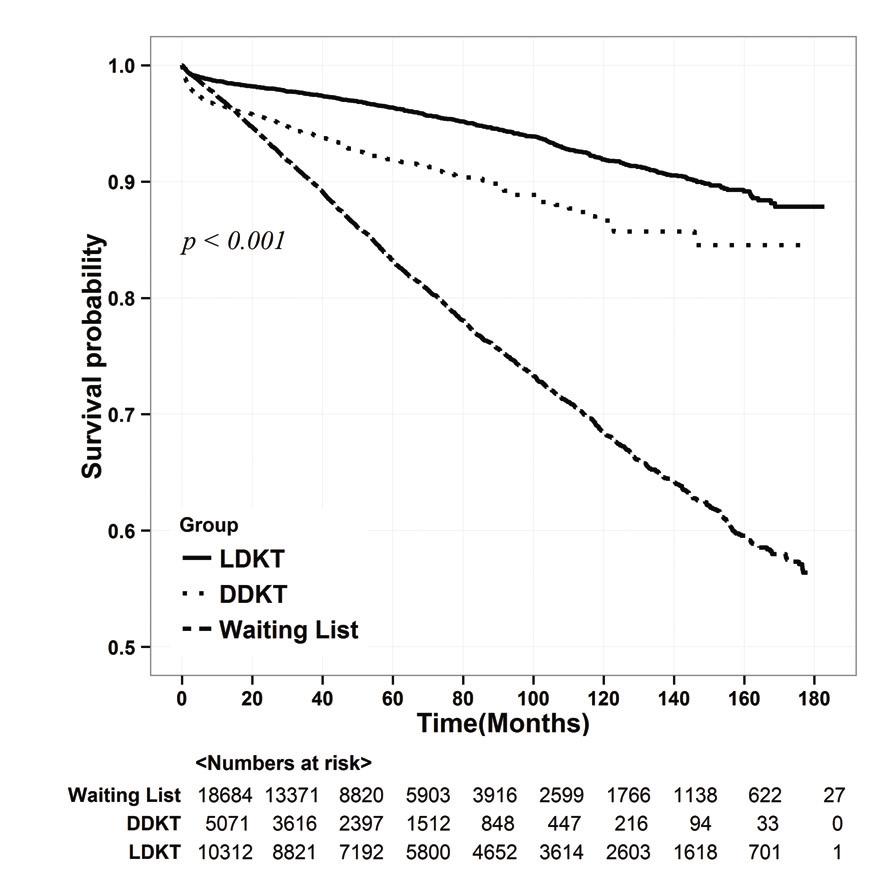


**Supplement Figure 2.** Comparison of the cumulative survival rate between waiting list patients using the Korean Network for Organ Sharing database (KONOS), matched-control group, and unmatched-control group in our study participants (data from the Korea Health Insurance Review and Assessment Service)


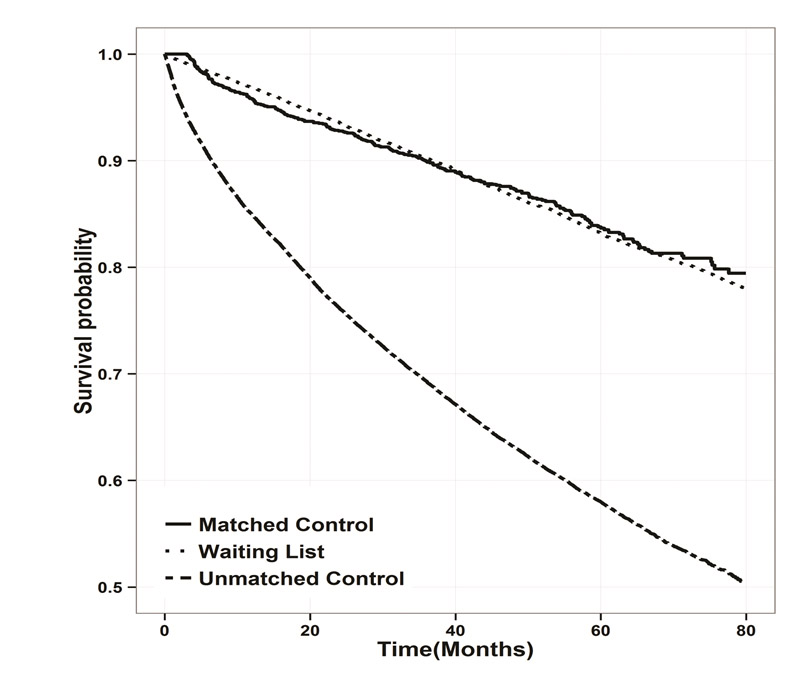


**Supplement Figure 3.** Comparison of MACE-free survival between CRC for ESRD cohort data and matched control group in our study participants (data from the Korea Health Insurance Review and Assessment Service)


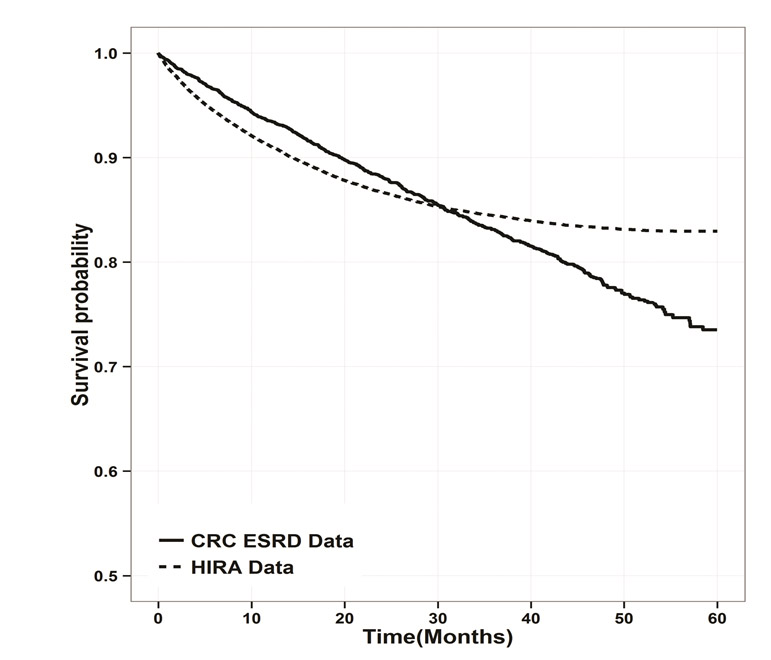

Supplement: Supplemental Digital Content [file medi-95-e4352-s001.doc]
